# Periodontopathic bacteria in rheumatoid arthritis pathogenesis: bridging clinical associations to molecular mechanisms

**DOI:** 10.3389/fimmu.2025.1681037

**Published:** 2025-10-17

**Authors:** Xiaojing Guo, Siwei Wang, Jiuli Ding, Weiwei Liu, Jiaqi Xu, Mutian Wang, Hongyuan Sun, Yuening Ma, Wei Liu, Lei Zhang, Min Liu

**Affiliations:** ^1^ Department of Infectious Diseases, First Teaching Hospital of Tianjin University of Traditional Chinese Medicine, Tianjin, China; ^2^ Department of Infectious Diseases, National Clinical Research Center for Chinese Medicine Acupuncture and Moxibustion, Tianjin, China; ^3^ Graduate School, Tianjin University of Traditional Chinese Medicine, Tianjin, China; ^4^ Department of Respiratory Medicine, Shanxi Provincial Academy of Traditional Chinese Medicine, Shanxi, China; ^5^ Office of the President,Tianjin Academy of Traditional Chinese Medicine Affiliated Hospital, Tianjin, China

**Keywords:** rheumatoid arthritis, periodontitis, *porphyromonas gingivalis*, citrullinated antigens, oral pathogens

## Abstract

Periodontitis (PD) is a chronic inflammatory disease linked to microbial dysbiosis, while rheumatoid arthritis (RA) is an autoimmune disorder characterized by anti-citrullinated protein antibodies (ACPA). Despite their distinct etiologies, a clinical and serological association between PD and RA has been observed. Oral microorganisms, especially *Porphyromonas gingivalis* (*P. gingivalis*), may contribute to RA onset or progression through dissemination to joints or systemic inflammation. This review explores a: the role of oral microbiota and immune responses in RA b; clinical pathogenic pathways from oral pathogens to the joints c; mechanistic studies on the impact of periodontal pathogens on RA; and d. preventive and therapeutic strategies. *P. gingivalis* and other periodontal pathogens have been detected in synovial tissues and fluids of RA patients. Microbiome analyses show a more diverse oral microbiota with elevated periodontal disease-associated bacteria in RA patients. Studies demonstrate that *P. gingivalis* can induce citrullination, autoantibody production, and inflammation, exacerbating joint damage. Future research should investigate the impact of periodontal therapy and RA treatments on the oral microbiota, while large-scale clinical trials are needed to validate the causal relationship between periodontal pathogens and RA.

## 1 Introduction

Rheumatoid arthritis (RA) has attracted widespread attention due to its substantial impact on human health and the considerable socioeconomic burden it imposes. It is commonly associated with progressive disability, premature mortality, and elevated healthcare and societal costs. The global prevalence of RA varies substantially, ranging from 0.25% to 1% (1), and is projected to continue rising through 2040. This trend poses a significant challenge to global public health (2). RA primarily affects individuals over the age of 40. The prevalence is particularly higher in women, who are two to three times more likely to be affected than men (3). RA is a systemic autoimmune inflammatory disease that primarily affects the joints. The pathological hallmark of RA is persistent synovitis, which leads to joint pain, cartilage degradation, joint deformity, and functional impairment. In advanced stages, RA can result in severe disability (4).

### 1.1 Risk factors for rheumatoid arthritis

**Figure 1:**
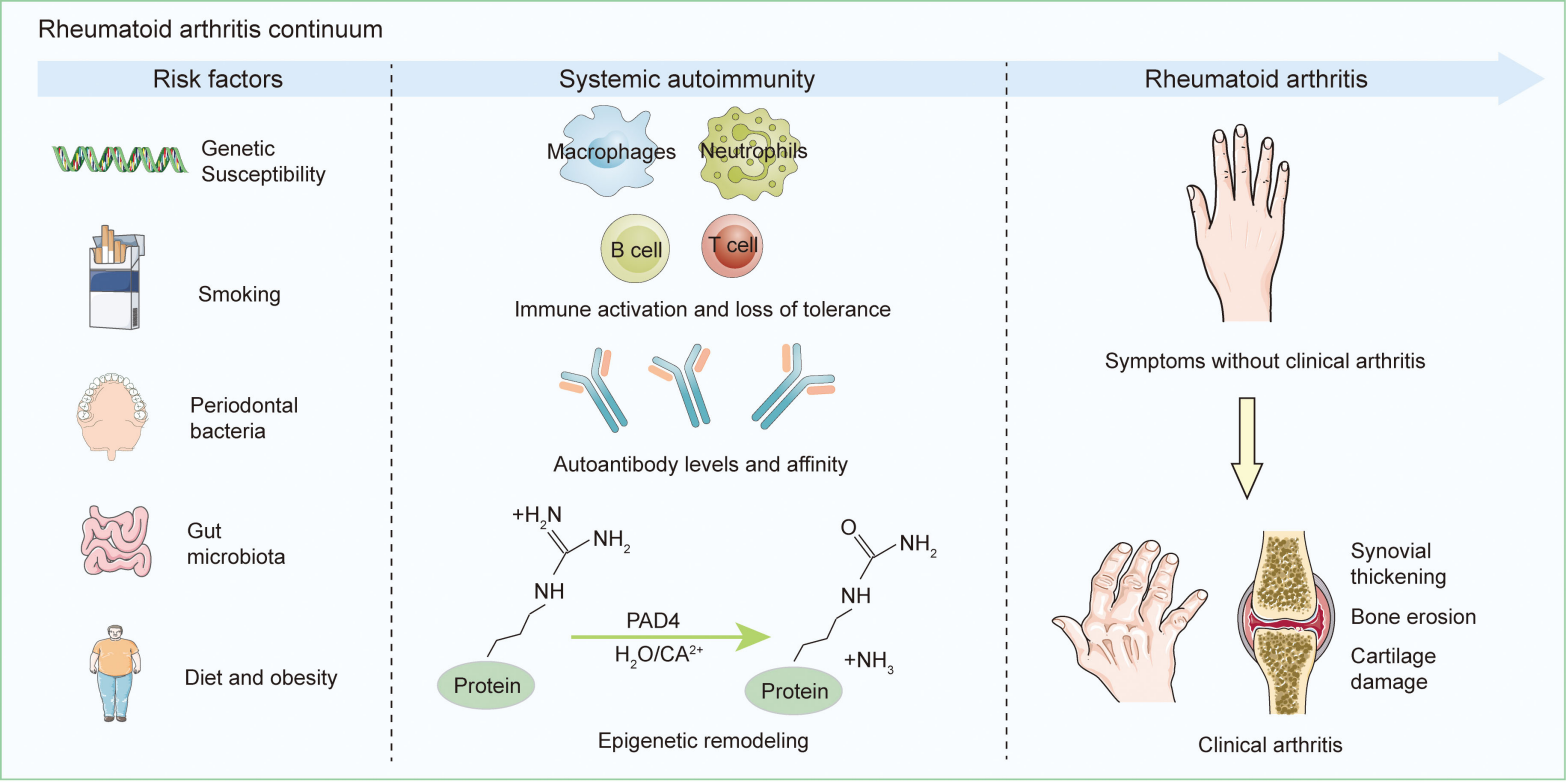
Initiation and Advancement of RA. A combination of individual genetic predispositions and environmental exposures contributes to systemic autoimmunity in those susceptible to RA, resulting in the localized synthesis of RA-related antibodies, predominantly in the oral, lung, or gut mucosal tissues. Many patients then progress through a stage of subclinical synovitis before developing RA. This process is not inevitable; ACPA positivity before synovitis may convert to ACPA negativity, and some ACPA-positive individuals may not develop RA.

The development of RA is influenced by both genetic and environmental risk factors (**Figure 1**). Genetic predisposition is estimated to account for approximately 60 percent of RA risk (5, 6). First-degree relatives of RA patients have a two- to fivefold higher risk compared to the general population. The genetic susceptibility to RA is strongly associated with specific *human leukocyte antigen* (*HLA*) haplotypes, particularly the presence of the shared epitope (*SE*), a five–amino acid motif encoded by certain *HLA-DRB1* alleles (7). Compelling evidence supports *HLA-DRB1* as the primary genetic determinant in anti-citrullinated protein antibodies(ACPA)-positive RA (8). Genome-wide association studies (GWAS) have further revealed the contribution of non-HLA genes to RA pathogenesis. The genetic risk of seropositive RA, defined by the presence of ACPA and/or rheumatoid factor, is primarily linked to signaling networks involving interferon alpha/beta and interleukin-12/23. Variants in genes within the JAK-STAT pathway, including STAT4, TYK2, and FLT3, have shown significant associations with disease susceptibility (9). Individuals who are ACPA-positive have a substantially increased risk of progressing to classified RA and are considered to be in an at-risk state (10), which is marked by immune dysregulation. Epigenetic studies have revealed nonspecific regulatory abnormalities in peripheral B cells, as well as in naïve and memory T cells of ACPA-positive individuals. Studies of antigen-specific T cells have identified a higher frequency of CD4^+^ T cells that recognize citrullinated epitopes, especially citrullinated cartilage intermediate layer protein I (cit-CILP), in at-risk individuals. These T cells tend to exhibit pro-inflammatory phenotypes, such as T helper cell (Th) 1, Th17, and stem-like memory characteristics. Additionally, antibody–antigen microarray analysis has detected increased levels of autoantibodies against citrullinated clusterin, fibrinogen, and histone H4 in both at-risk individuals and patients with early RA, with the highest levels found in the latter (10). These findings suggest that widespread early epigenetic alterations may disrupt immune tolerance and lead to the activation of cit-CILP–specific T cells, which in turn support B cell responses and drive the production of autoantibodies against a broader array of citrullinated antigens. This multifaceted immune imbalance promotes the progression from asymptomatic autoimmunity to clinically evident arthritis.

An increasing number of environmental factors have been identified as contributing to the risk of developing RA. Among them, cigarette smoking is currently recognized as the most strongly associated environmental risk factor for seropositive RA, with a clear dose-dependent effect (11). Compared to non-smokers, RA patients who carry *SE* alleles and smoke exhibit elevated ACPA production by peripheral B cells (12). Further studies indicate that smoking and *HLA-SE* contribute at distinct stages of RA development. Smoking promotes the generation of ACPA and the onset of symptoms, while *HLA-SE* primarily facilitates the emergence of clinical manifestations and progression to classified RA (13). The role of the microbiome in RA has also garnered growing interest, and the involvement of oral and gut microbial communities is now well recognized. Studies suggest that mucosal exposure and/or dysbiosis in patients with early RA (diagnosed within one year) or long-standing active disease may play a causal role in the at-risk phase of RA progression (14). Periodontal dysbiosis may induce local inflammation, disrupt mucosal barriers, and allow microbial components to translocate into the bloodstream. Certain bacterial elements may also mimic host autoantigens at the molecular level, potentially triggering joint-specific immune responses (15). Overweight and obesity, defined as a body mass index (BMI) of ≥25 kg/m^2^ and ≥30 kg/m^2^, respectively, have also been linked to increased RA risk (16). Studies have shown that individuals with a BMI ≥25 kg/m^2^ and ≥30 kg/m^2^ experience a 15% and 21–31% greater risk of developing RA, respectively (17). This association may be mediated by the pro-inflammatory activity of white adipose tissue and its immunomodulatory effects (18). Nevertheless, the role of obesity in RA onset remains controversial. Other potential environmental risk factors, such as dietary habits and estrogen levels, have been proposed but require further investigation to confirm their relevance (1). Overall, most known environmental contributors to RA point toward the involvement of chronic mucosal inflammation in disease initiation, although additional research is required to clarify the underlying mechanisms (1).

### 1.2 The pivotal role of citrullination in RA pathogenesis

The pathogenesis of RA is complex. Immune dysregulation may begin years before the appearance of joint symptoms, a period commonly referred to as the preclinical phase of RA (19). The formation and recognition of citrullinated self-antigens span the entire course of the disease, from the subclinical phase to the clinical manifestation (20). This process is primarily attributed to the loss of immune tolerance, resulting in aberrant immune responses against citrullinated self-antigens (21). Citrullination is a key post-translational modification in which arginine residues in specific self-antigens, such as immunoglobulin G (IgG), type II collagen, and vimentin, are converted into citrulline by peptidyl arginine deiminases (PADs) (22). Among the five calcium-dependent PAD isoenzymes in humans (23), PAD2 and PAD4 are most strongly associated with RA. These enzymes are highly expressed in synovial tissue, synovial fluid, and infiltrating mononuclear cells, with expression levels positively correlated with the severity of inflammation (24, 25). Citrullinated proteins have also been abundantly detected in synovial fluid, where they serve as key targets for local immune responses (26–28). Citrullination exposes cryptic epitopes that are normally hidden from immune surveillance, leading to their misrecognition as non-self antigens. This is achieved by altering protease cleavage sites and destabilizing protein structures, which unmasks peptides derived from native sequences (29). In the presence of susceptibility alleles such as *HLA-DR1* and *HLA-DR4*, the positively charged P4 pocket of the *SE* binding groove selectively binds citrullinated peptides through electrostatic complementarity. These complexes are misrecognized as foreign, triggering downstream immune cascades (30). Citrullinated antigens are taken up by antigen-presenting cells, which initiate dendritic cell-mediated responses and activate both T and B cells in a process known as costimulation. B cells undergo somatic hypermutation and class-switch recombination, eventually differentiating into plasma cells that produce specific autoantibodies against citrullinated epitopes, known as ACPAs (31). ACPAs can be detected in the bloodstream approximately four to five years before the clinical onset of arthritis. As genetic and environmental risk factors interact, ACPA levels increase, the range of recognized protein epitopes expands, and circulating pro-inflammatory proteins rise (20). These changes collectively contribute to the breakdown of immune tolerance and the eventual transition to clinically evident arthritis, whether classified or undifferentiated (29).

Increasing evidence supports the mucosal origin hypothesis in the pathogenesis of RA, suggesting that autoimmune processes may begin at extra-articular mucosal sites—such as the lungs, oral cavity, and gut—where local immunological changes lead to both local and systemic autoantibody production (14). Among these sites, the oral mucosa, particularly in the setting of PD, has garnered significant attention. A meta-analysis demonstrated that individuals with PD lasting more than five years are significantly more likely to develop RA, with a 69% higher risk than healthy controls (32). Citrullination may initiate autoimmune responses at periodontal sites before the clinical onset of RA (33, 34). PAD2, PAD4, and ACPAs have been detected in inflamed gingival tissues and gingival crevicular fluid in individuals with PD (35). ACPAs targeting citrullinated peptides are among the most extensively studied autoantibodies in RA. Their targets include intracellular proteins such as α-enolase and vimentin, as well as extracellular citrullinated proteins such as fibrinogen, type II collagen, and fibronectin (36, 37). These antigen–antibody complexes accumulate in synovial fluid and contribute to RA pathogenesis (38). *Porphyromonas gingivalis* (*P. gingivalis*), a key periodontal pathogen, produces peptidyl arginine deiminase (PPAD), an enzyme that citrullinates arginine residues in host periodontal proteins and its own bacterial proteins. Arginine-specific gingipains (Rgps), another major virulence factor of *P. gingivalis*, cleaves fibrinogen and α-enolase to generate C-terminal arginine-containing peptides, which serve as preferred substrates for PPAD (39, 40). Co-incubation of these proteins with PPAD and RgpB has confirmed the formation of novel citrullinated peptides, including 37 derived from fibrinogen and 11 from α-enolase (41).Rheumatoid factors (RFs), another class of autoantibodies in RA, target the Fc region of host IgG (42). Rgps cleave lysine and arginine residues within the CH2 and CH3 domains of IgG, producing fragments that enhance Rheumatoid factor (RF) binding. Moreover, *P. gingivalis* generates short-chain fatty acids that attract neutrophils to periodontal tissues (43), where they activate and release reactive oxygen species (ROS) such as superoxide, hydrogen peroxide, and hydroxyl radicals. These ROS enhance the reactivity of IgG, facilitating RF-mediated immune complex formation (44). The resulting immune complexes involving ACPAs and RFs accumulate in synovial fluid, where they drive persistent synovitis and joint destruction—the pathological hallmarks of RA (38, 42). Other oral pathogens that may contribute to citrullination include *Aggregatibacter actinomycetemcomitans* (*A. actinomycetemcomitans*) *(* 45) and *Prevotella intermedia* (*P. intermedia*) *(* 46), which will be further discussed in subsequent sections.

### 1.3 Epidemiological association RA– periodontal disease

Periodontal disease affects the gums, supporting connective tissues, and alveolar bone, ranking sixth in disease burden among chronic non-communicable diseases. Between 2011 and 2020, its prevalence reached nearly 62% among adults who had received dental treatment (47). In 2021, over 1 billion people worldwide suffered from severe periodontitis, accounting for approximately 11% of the adult population (48). It is broadly classified into gingivitis and PD. Gingivitis is characterized by inflammation limited to the gingival epithelium and adjacent soft connective tissues, whereas periodontitis is a chronic, non-communicable, and multifactorial inflammatory condition primarily initiated by bacterial infection, leading to inflammation of the tooth-supporting structures (49). This process results in the loss of connective tissue attachment and alveolar bone destruction, with an overall population prevalence estimated at 45 to 50 percent. The pathogenesis of periodontitis involves the invasion of periodontal tissues by pathogenic bacteria and their products within the dental biofilm, which stimulate the production of pro-inflammatory mediators. Recurrent disruption of the oral mucosal barrier facilitates the translocation of oral pathogens and their virulence factors into the bloodstream (50). This leads to elevated systemic levels of inflammatory cytokines and a sustained immune-inflammatory response, which may serve as a persistent driver of systemic immune activation.

A growing body of clinical and epidemiological evidence supports a bidirectional relationship between RA and PD (51). The prevalence of PD among individuals with RA is at least twice as high as that observed in healthy populations (52). A study by Eriksson et al. reported that approximately 75 percent of RA patients exhibited moderate to severe periodontitis, while the remaining patients had either no periodontitis or only mild forms (53). A meta-analysis further revealed that individuals with RA display significantly worse periodontal conditions, characterized by greater clinical attachment loss (CAL), higher rates of tooth loss, elevated plaque index scores, and increased probing depth (54). These differences appear to be independent of age, sex, ethnicity, and smoking status (55). Notably, RA patients with severe periodontitis tend to exhibit higher levels of disease activity, highlighting a potential link between periodontal burden and RA progression (56).

## 2 Oral microorganisms in RA

The oral microbiota is a highly diverse and structured microbial community comprising over 700 bacterial species, including more than 250 recognized pathogens, which can influence host pathophysiology on a systemic level (57). Factors such as aging, poor oral hygiene, systemic comorbidities, and the use of medications can disrupt the composition of the oral microbiota, contributing to the onset of oral diseases (58). Over the past two decades, a growing number of clinical studies have examined the oral microbial characteristics of individuals at high risk of developing RA and of patients already diagnosed with the condition (summary provided in **Table 1**), revealing a potential link between oral microbial dysbiosis and RA pathogenesis.

**Table 1:** Studies on oral microbiota in RA patients.

Author, country, year	Inclusion criteria	Exclusion criteria	Study: design, number of participants, groups	Samples, microbiological methods	Results	Ref.
Kroese et al, The Netherlands, 2021	Early RA with diagnosed<1 year, or high-risk individuals with inflammatory arthralgia and elevated RF and/or ACPA levels; healthy controls without autoimmune conditions; all participators age≥18 years, and had a minimum of 12 natural teeth	None	Individuals at high-risk for RA(n=50), RA patients(n=50) and healthy(n=50)	The subgingival plaque, saliva, and tongue by 16S ribosomal DNA amplicon sequencing	Early RA patients and at-risk individuals had similar oral microbiomes, with higher abundances of *Prevotella* in saliva and *Veillonella* in saliva and tongue coating compared to healthy controls	(59)
Tong et al, China, 2019	Individuals at high-risk for RA with ACPA^+^, with or without arthralgia; RA patients were diagnosed; healthy controls with no history of inflammatory arthritis and ACPA^-^; all groups were≥18 years old	Having a history of antibiotics treatment or surgery in the last 3 months, current extreme diet, major organ dysfunction, cancer, other rheumatic or autoimmune diseases, and received biological agents	Case-control study, individuals at high-risk for RA(n=29), RA patients(n=27) and healthy(n=23)	Saliva, 16S rRNA gene amplicon sequencing	In pre-clinical RA, salivary microbial diversity was reduced, with decreased *Defluviitaleaceae_UCG-011* and *Neisseria oralis*, increased *Prevotella_6*	(60)
Mankia et al, UK, 2019	Early RA with anti-CCP^+^ and received DMARD therapy within the first 3 months; CCP^+^ at risk individuals with new-onset musculoskeletal symptoms but no clinical synovitis; healthy controls had no joint disease or history of inflammatory arthritis	None	Cross-sectional study, anti-CCP^+^ individuals without arthritis(n=48), early RA patients (n=26), healthy controls (n=32)	Subgingival plaque by shotgun metagenomic	Anti-CCP^+^ at-risk individuals have dysbiotic subgingival microbiomes with increased *P. gingivalis*, while the early RA group shows lower *P. gingivalis* abundance compared to controls	(64)
Cheng et al, UK, 2021						(62)
Arleevskaya et al, Russia, 2022	Early RA was diagnosed and with development <1 year from diagnosis; RA patients were diagnosed; non-RA individuals enriched in first-degree relatives from RA probands and clinically suspect arthralgia was established when the score was ≥3	None	Cross-sectional cohorts, women individuals with early RA(n=15), women individuals with first-degree relatives from RA probands (n=69), women with established RA(n=42)	Oral mucosa by PCR for 16S rRNA genes	A decrease in *Porphyromonas* spp. and *Aggregatibacter* spp. correlates with elevated ACPA levels in RA patients	(61)
Kim et al, Republic of Korea, 2022	Pre-RA subjects had positive RF and/or ACPA, but did not definite synovitis; new-onset RA lasting less than 6 months; chronic RA lasting over 6 months	Antibiotic use within 3 months, as well as any medical conditions that required antibiotic use	Case-control study, patients of preclinical RA (n=5), new-onset RA (n=2), and chronic RA(n=3), matched control subjects(n=72)	Subgingival plaque, 16S rRNA sequencing	Preclinical RA patients had higher subgingival *Porphyromonadaceae* abundance than new-onset or chronic RA, with distinct microbial communities in high-positive RF versus low/negative RF patients	(63)
Wolff et al, Germany, 2014	RA patients had to have active RA active who defined as DAS28 >3.2; early RA with symptom onset <2 years; all patients had to be otherwise healthy, Caucasian, and aged between 30 and 75 years	Therapies interfering with oral hygiene, systemic disease-related periodontitis, periodontal therapy in the past 5 years, recent professional tooth cleaning, antibiotic intake, or pregnancy/nursing in the past 6 months.	Cross-sectional study, patients with early RA(n=22), healthy controls (n=22)	Supragingival and subgingival biofilm, real-time quantitative PCR	*Tannerella forsythia* subgingivally and *Streptococcus anginosus* supragingivally are characteristic pathogens of early RA	(66)
Esberg et al, Sweden, 2021	Early-onset RA with symptom duration ≤ 12 months and who fulfilled the 1987 ACR classification criteria	Had taken antibiotics during the preceding 3 months, DMARD treatment or any other known autoimmune condition	Case-control study, early onset of RA patients (n=61), healthy individuals (n=59)	Saliva, 16S rRNA gene amplicon sequencing	Microbiota comparison in early RA patients revealed distinct diversity and notable enrichments of *Filifactor alocis*, *Porphyromonas endodontalis*, *Prevotella pleuritidis* and *Treponema denticola*.	(65)
Kozhakhmetov et al, Kazakhstan, 2023	Established RA diagnosis > 1 year	Pregnancy, lactation, oncology, comorbid conditions, decompensated chronic diseases, antibiotics/probiotics use (last 3 months); healthy controls had heredity for autoimmune diseases	Case-sectional study, RA patients in female(n=75) and healthy volunteers (n=114)	Scrap from tongue, gums, tonsils, and plaque, 16S rRNA gene amplicon sequencing	RA patients' oral samples showed higher bacterial diversity, increased *Prevotellaceae* and *Leptotrichiaceae*, but lower butyrate and propionate-producing bacteria	(67)
Eriksson et al, Sweden, 2019	Diagnosis of RA	Pregnancy, lactation, other forms of arthritis, recent antibiotic use within the last 3 months, and any periodontal treatment received at least 3 months prior to the dental examination	RA patients(n=40)	Saliva, gingival crevicular fluid and subgingival plaque, by16S rRNA Sequencing and qPCR	Moderate/severe PD showed higher abundances of *Desulfobulbus* sp., *Prevotella* sp., *NA* sp., *Bulleidia* sp., *Capnocytophaga* sp., and *Tannerella forsythia*, while *Prevotella oris* and a *Porphyromonas* sp. were more abundant in no/mild periodontitis.	(53)
Beyer et al, Norway, 2018	Diagnosis of RA, caucasian ethnicity and ≥ 35 years of age	Diabetes, malignancy, pregnancy, breastfeeding and antibiotic use within 3 months prior to the study	Cross-sectional study, RA patients(n=78)	Subgingival plaque, 16S rDNA qPCR, 16S rDNA amplicon sequencing	Active RA patients have better oral health and a healthier subgingival microbiome compared to those in remission	(68)
Ziebolz et al, Germany, 2011	RA patients were aged 18 to 70 years	Additional general disorders and diseases, metabolic disorders, infectious diseases, seizure disorders or neuropathy, addictive disorders, renal disorders and organ transplants, pregnancy, and the patients had no therapy for PD up until the time of the study	RA patients(n=66)	Subgingival plaque, PCR	Most RA patients had moderate-to-severe periodontitis with prevalent periodontal pathogens. *Fusobacterium nucleatum*, *Eikenella corrodens*, and *Parvimonas micra* were more common, and *A. actinomycetemcomitans*(16%), *P. gingivalis* (58%) and *Tannerella forsythia*(78%) were less common	(72)
Corrêa et al, Brazil, 2019	RA patients and presence of at least 8 teeth	Other rheumatic disease, treatment for periodontal disease within the last 6 months, using of orthodontic appliances, using of antibiotics within the last 3 months, pregnancy and lactation	Cross-sectional study, RA patients (n=42), individuals without RA or other rheumatic diseases(n=47)	Subgingival plaque, 16S rRNA gene amplicon sequencing and qPCR	RA patients, even without periodontitis, exhibited an enrichment of periodontitis-associated bacteria, including *Prevotella* species, and a reduction in health-associated species.	(73)
Chen et al, USA, 2022	RA patients	smokers, those with malignancy or pregnancy, those who were breastfeeding, and those who were under antibiotic treatment within fewer than three months prior to the enrollment	RA patients(n=30), controls(n =25)	Subgingival plaque, 16S rRNA gene amplicon sequencing	In RA patients, *Streptococcus anginosus*, *Treponema denticola*, and an unidentified *Fusobacterium* species were enriched, along with RA-exclusive *A. butyrica* and *P. simiae*.	(74)
Lehenaff et al, USA, 2021	RA who were naïve to biologic therapy	1)<18 years, 2)<8 natural teeth, 3) antibiotic treatment in the three months preceding, 4) immune compromising conditions, 5) using dental products one week prior or during the study period	RA patients(n=8), non-RA subjects(n=10)	Subgingival plaque from both shallow and deep subgingival sites, 16S rRNA gene amplicon sequencing	In RA patients, the microbiomes of deep and shallow sites were more similar, with overabundant *Streptococcus parasanguinis* and *Actinomyces meyeri*.	(69)
Schmickler et al, Germany, 2017	Aged 18 to 70 years with diagnosis of RA	General disorders interfering with oral examinations, additional immunosuppressive therapy, participants have been treated with antibiotic therapy within the last 3 months	Cross-sectional study, RA patients(n=168) healthy control(n=168)	Subgingival biofilm, PCR	RA patients had worse periodontal conditions than healthy controls, and aCCP-positive patients showed higher *F. nucleatum* and *P. gingivalis* concentrations.	(52)
Lopez-Oliva et al, UK, 2018	patients with diagnosed RA	None	Case-control study, RA patients(n=22), non-RA controls (n=19)	Subgingival plaque, 16S rDNA sequencing	The oral microbiome in RA is enriched with inflammophilic and citrulline-producing organisms, especially *Cryptobacterium curtum*, along with species from *Actinomyces*, *Dialister*, *Desulfovibrio*, *Fretibacterium*, *Leptotrichia*, *Prevotella*, *Selenomonas*, *Treponema*, and *Veillonellaceae*.	(71)
Cheah et al, Malaysia, 2022	Patients>25 years old, presented with at least twelve teeth, and fulfilled the classification of RA	Non-malaysians, had taken antibiotics, and/or periodontal therapy during the previous 4 months before the study, who were pregnant or had concurrent systemic or debilitating conditions	Cross-sectional comparative study, RA patients(n=49), non-RA patients (n=51)	Subgingival plaque, 16S rRNA sequencing	RA patients demonstrated subgingival microbial dysbiosis featured networks with health- and disease-associated genera. In the RAwith periodontal healthy network, the key genera were *Rothia*, *Capnocytophaga*, and *Alloprevotella*, while in the RAwith PD network, they were *Capnocytophaga*, *Corynebacterium*, and *Fretibacterium*.	(75)
Zhang et al, China, 2015	Individuals with RA between 18 and 65 years old with a disease duration of at least 6 weeks, at least one swollen joint and at least three tender joints were enlisted	Had a history of chronic serious infection, any current infection or any type of cancer, pregnant or lactating women	Dental plaques (n = 105) and saliva (n = 98) from RA and healthy individuals	Dental and saliva, metagenomic shotgun sequencing	Gut and oral microbiome concordance showed dysbiosis in RA patients, partially resolved after treatment. *Haemophilus* spp. were depleted, whereas *Lactobacillus salivarius* was over-represented in individuals with RA	(76)
Eriksson et al, Sweden, 2022	Patients with diagnosed RA	Had received periodontal treatment in the past 3 months, had other forms of arthritis, used antibiotics within 3 months before the examination, or were pregnant or lactating	RA patients with chronic PD(n=53), non-RA with chronic PD(n=48)	Saliva, 16S rRNA sequencing	*Granulicatella* sp., *Veillonella* sp., *Megasphaera* sp., and *Fusobacterium nucleatum* were the most highly enriched in the RA group	(77)
de Jesus et al, Canada, 2021	Patients with diagnosed RA	None	Case-control study, RA patients(n=35) and non-RA controls(n=64)	Oral mucosa samples by Illumina MiSeq sequencing	*Streptococcus salivarius*, *Rothia mucilaginosa, Prevotella* sp., *Leptotrichia* sp., and *Selenomonas fueggei* were more abundant in RA	(78)
Mikuls et al, USA, 2018	RA patients with an age of disease onset >18 years; OA patients was based on medical documentation or imaging results consistent with degenerative arthritis	None	Multicenter case–control investigation, RA patients(n=260), OA patients(n=296)	Subgingival plaque, 16S rRNA gene amplicon sequencing	Subgingival microbiome profiles in RA patients are similar to those in OA patients, but accounting for periodontitis, 10 taxa, including *Catonella* sp., *Clostridiales* sp., and *Porphyromonas* sp., are less abundant in RA.	(70)

DAS28: disease activity score in 28 joints.

### 2.1 Oral microbiota

Individuals at high risk of RA are characterized by seropositivity for autoantibodies and inflammatory arthralgia in the absence of clinically evident synovitis (59–64). Oral microbiota perturbations have been observed during this “preclinical” phase of RA. Studies have demonstrated a reduction in salivary microbial diversity among these high-risk individuals, particularly a decrease in *Defluviitaleaceae_UCG-011* and *Neisseria oralis*, along with an increase in *Prevotella_6 (* 60). Dysbiosis of the subgingival microbiota has also been detected in anti-cyclic citrullinated peptide (CCP)^+^ individuals without arthritis, who show higher prevalence of both periodontitis and gingivitis (62, 64). Furthermore, the oral microbiota composition in at-risk individuals closely resembles that of early RA patients, with no significant differences observed in the microbial profiles of saliva and tongue coating. Compared to healthy controls, these groups exhibit increased abundance of *Veillonella* in both saliva and tongue coating, and *Prevotella* in saliva (59). This similarity suggests that specific shifts in the oral microbiota may constitute early pathogenic events in RA development. Additionally, studies have identified enrichment of *Porphyromonadaceae* family bacteria and *Saccharimonas* species in subgingival plaque samples from at-risk individuals prior to RA diagnosis (63). These microbial signatures may hold predictive potential but require further confirmation in prospective investigations.

To investigate the oral microbiota across different stages of RA, patients are commonly categorized into early-stage and chronic RA, with healthy individuals serving as controls (61–66). Early RA is typically defined as meeting the 2010 ACR/EULAR classification criteria, with disease duration under 6 months (63), less than one year since diagnosis (61, 65), initiation of disease-modifying antirheumatic drugs (DMARDs) therapy within 3 months (62, 64), or symptom onset within two years (66). Compared to healthy individuals, early RA patients tend to experience more severe periodontitis, including greater loss of periodontal attachment and alveolar bone. Subgingival Tannerella forsythia and supragingival Streptococcus anginosus are markedly elevated—by sixfold and threefold, respectively—indicating their potential as characteristic pathogens in early RA (66). The composition of the oral microbiota in RA patients shifts in accordance with the severity of coexisting periodontitis. In individuals with no or mild periodontitis, *Prevotella oris* and *Porphyromonas* species are more abundant, whereas in moderate to severe cases, the dominant species include *Desulfobulbus* sp.*, Prevotella* sp., *NA* sp., *Bulleidia* sp., and *Capnocytophaga* sp., along with higher levels of *Tannerella forsythia (* 53). This indicates that the hypoxic and inflamed periodontal microenvironment remains a critical determinant of subgingival microbial composition in the context of RA. Distinct oral microbial signatures have also been identified based on RA disease activity. *Staphylococcus* and *Porphyromonas* are associated with higher disease activity, while *Treponema* sp. and *Absconditabacteriales* are more frequently found in patients during remission (67). A seemingly paradoxical yet significant observation is that patients with active RA sometimes exhibit better periodontal health and a more “eubiotic” subgingival microbiota compared to those in remission (68). This strongly suggests that anti-rheumatic treatments may substantially alter the oral microbial landscape and inflammatory status, necessitating careful consideration of therapy as a major confounding factor when interpreting microbiota–disease activity relationships. Oral microbiota may also correlate with the presence and titers of RA-specific autoantibodies. Marked differences in subgingival microbial communities have been observed between RF-high and RF-negative/low patients, with enrichment of *Fusobacteria*, *Saccharibacteria*, and *Spirochaetes*, as well as the families *Saccharimonas*, *Porphyromonadaceae*, and *Spirochaetaceae*, associated with elevated RF levels (63). Similarly, ACPA positivity has been linked to more severe PD (53). Certain taxa, such as *Prevotella_9*, show a positive correlation with both ACPA and RF levels (67), whereas reductions in *Porphyromonas* and *Aggregatibacter* have been associated with higher ACPA titers, independent of RF status, *HLA-DRB1 SE* alleles, or methotrexate usage (61). This may reflect a unique host–microbe interaction in which ACPA targets citrullinated bacterial membrane antigens, potentially facilitating the clearance of specific pathogens both locally and systemically. Notably, no significant microbial differences have been found between superficial and deep gingival sites in RA patients (69). Moreover, subgingival microbiota profiles in RA patients resemble those of individuals with osteoarthritis (OA), and microbial diversity indices do not correlate with RA disease activity, suggesting that microbial dysbiosis in the subgingival niche is not exclusive to RA (70). Overall, RA patients exhibit higher total bacterial loads and a more diverse oral microbiota, with increased prevalence of proinflammatory and citrulline-producing organisms (71), while the abundance of health-associated commensals is concurrently reduced (67, 72, 73).

### 2.2 Periodontal pathogen distribution in oral niches and clinical correlates in RA patients

Although numerous studies have highlighted the alterations in oral microbiota associated with different stages of RA, disease activity levels, and autoantibody profiles, it is important to consider the complexity of the oral environment. The oral cavity consists of distinct ecological niches that support heterogeneous microbial communities. Microbial composition differs significantly between sites such as dental plaque, the dorsal tongue, and keratinized gingiva (79). Within this complex microenvironment, *P. gingivalis* has become a central focus of mechanistic research due to its unique ability to generate citrullinated proteins and its strong epidemiological association with RA, as discussed in Section 1.3. As a Gram-negative anaerobe, *P. gingivalis* is commonly found in individuals with poor oral hygiene and has also been implicated in several systemic diseases. Meta-analyses have demonstrated that individuals exposed to *P. gingivalis* have a significantly increased risk of developing RA (80). In ACPA^+^ individuals at high risk of RA, *P. gingivalis* shows increased abundance in subgingival plaque (62, 64), but reduced levels in oral mucosal surfaces and saliva (60, 61). A large cross-sectional study involving 600 participants found that elevated *P. gingivalis* DNA levels in saliva were associated with increased serum ACPA titers (81), suggesting the potential for systemic dissemination. Furthermore, in participants with concurrently elevated salivary *P. gingivalis* DNA and C-reactive protein (CRP), ACPA reactivity against citrullinated α-enolase and vimentin was commonly observed (82). Interestingly, other studies have reported no significant difference in the prevalence of *P. gingivalis* in subgingival plaque between RA patients and healthy controls (83–85). Additionally, *P. gingivalis* abundance has shown no consistent association with RA disease activity, ACPA, or RF levels (68, 70, 81, 86), but instead correlates more strongly with PD severity. These findings suggest that PD, rather than *P. gingivalis* per se, may be the primary factor modulating RA outcomes. Nonetheless, this does not negate the potential involvement of *P. gingivalis* in RA. In tongue biofilm samples, the proportion of *P. gingivalis* has been positively correlated with RA disease activity, with higher detection rates in non-remission patients compared to those in remission (83). Although findings vary depending on the sample type, existing evidence supports the participation of *P. gingivalis* in the pathogenesis of RA. Beyond *P. gingivalis*, other periodontal pathogens have also been implicated in RA. *A. actinomycetemcomitans* is more frequently detected in the gingival crevicular fluid of RA patients (87), and its subgingival presence is positively correlated with DAS28, RF, anti-CCP, and ACPA levels (88). In addition, *Staphylococcus aureus* has been found at higher prevalence in the oral cavities of RA patients compared to controls (89, 90), indicating that multiple oral microbes may contribute to RA-related immune dysregulation.

Substantial evidence indicates that individuals at high risk for RA and RA patients often show characteristic oral microbiome dysbiosis, including reduced microbial diversity and altered abundance of various bacterial taxa. These changes have been linked to autoantibody presence, especially ACPA, as well as disease stage and activity. However, most studies are cross-sectional, limiting conclusions about causality and temporal sequence. Small sample sizes (n < 100), limited geographic scope (mainly Europe and East Asia), and inconsistent sampling sites further constrain generalizability. Although some studies control for confounders like smoking, age, sex, and medication use, fully eliminating these factors is difficult, particularly given the frequent co-occurrence of periodontitis and RA. Thus, observed microbial changes may partly reflect confounding rather than RA-specific patterns. Identifying reliable microbial biomarkers for RA diagnosis or prediction remains challenging. Future research should adopt large-scale, standardized, prospective designs and evaluate microbiome-targeted interventions for RA prevention and treatment. The next section discusses the role of periodontal pathogens in RA pathogenesis.

## 3 Pathogenic translocation of oral microbiota to synovial compartments

Oral pathogens possess the capacity to translocate from their primary infection sites to distant local tissues, including the joints. Several clinical studies in patients with RA (**Table 2**) have identified nucleic acids from periodontal pathogens within synovial fluid and synovial tissues, lending support to the hypothesis that oral bacteria may reach the joint environment and participate in the initiation and progression of arthritis. In a pioneering study, Moen et al. reported a greater diversity and higher concentration of oral bacterial DNA in synovial fluid compared to serum samples from patients with active RA. Notably, multiple periodontal pathogens such as *Tannerella forsythensis*, *P. intermedia*, *Streptococcus intermedius*, *P. gingivalis*, *A. actinomycetemcomitans*, and *Fusobacterium nucleatum* were detected (91), preliminarily confirming that oral bacteria can disseminate into the joint cavity. Subsequent studies have concentrated on specific pathogenic species. Reichert et al. demonstrated that the detection rate of *P. gingivalis* DNA was significantly higher in both the synovial fluid and oral plaque of RA patients compared to non-RA controls (92), suggesting preferential enrichment of this pathogen within the RA-affected joint environment. Further investigations revealed that *P. gingivalis* DNA was present not only in synovial fluid but also in synovial tissue, with a significantly higher positivity rate in RA patients (33.3%) than in those with undifferentiated polyarthritis (5.9%) (93). Moreover, the positivity rate in synovial tissue was particularly elevated in patients carrying the HLA-DRB1*04 allele, implying a potential interaction between bacterial localization and genetic susceptibility (93). However, it is important to note that the detection rates of *P. gingivalis* in subgingival plaque, peripheral blood, and synovial fluid did not significantly differ between patients with RA and those with undifferentiated arthritis (93). Furthermore, the presence of *P. gingivalis* was not significantly correlated with disease activity, disability levels, or autoantibody status in RA patients. In patients with refractory RA complicated by periodontitis, *P. intermedia* and *P. gingivalis* DNA were frequently detected in subgingival plaque and synovial fluid, with detection rates of 73.6% and 42.1%, respectively (94). These findings further support the hypothesis that specific periodontal bacteria may colonize the joint space, particularly in patients with both systemic and oral inflammatory conditions. Further insights into the joint microbiome have been gained from a study conducted in China, which found abundant bacterial nucleic acids in both synovial fluid and synovial tissue samples from RA patients. Among periodontal species, *Porphyromonas* was consistently detected in all samples, while *Veillonella dispar*, *Haemophilus parainfluenzae*, *Prevotella copri*, and *Treponema amylovorum* were more prevalent in synovial fluid. In contrast, genera such as *Agrobacterium*, *Comamonas*, *Kocuria*, *Meiothermus*, and *Rhodoplanes* were more abundant in synovial tissue (95), revealing the complex microbial composition within the RA joint microenvironment. Oral *streptococci* are dominant commensals in the human oral and pharyngeal cavities, and their bacterial fragments have also been identified in the joint spaces of RA patients (96). Animal models further corroborate the dissemination of periodontal pathogens. *P. gingivalis*, Treponema denticola, and Tannerella forsythia are recognized as periodontal pathogens that typically emerge in the mature stages of biofilm development. This microbial consortium, known as the red complex, constitutes a keystone polybacterial community within the subgingival biofilm of progressive periodontitis sites (97). Among members of the red complex, only *P. gingivalis* was found to disseminate hematogenously to synovial joints. Fluorescence *in situ* hybridization confirmed its presence in the perinuclear region of ankle joint cells in mice (98). Additionally, *streptococcal* cell walls extracted from the oral cavity of RA patients have been shown to induce arthritis in murine models. Local gingival inflammation and barrier dysfunction may facilitate the translocation of pro-inflammatory streptococcal components into the synovium, especially given the limited degradation capacity for such pathogen-associated molecular patterns (99).

**Table 2:** Studies detecting oral bacteria in synovial fluid/tissue of RA patients.

Author, country, year	Inclusion criteria	Exclusion criteria	Study: design, number of participants, groups	Samples, microbiological methods	Results	Ref.
Martinez-Martinez et al, Mexico, 2009	RA patients were under two or more DMARDs, most of them with non-steroidal anti-inflammatory drugs and low doses of steroids	pregnant and breastfeeding women, patients who having a history of antibiotics treatment in the last 3 months, and having previous treatment of PD	Cross-sectional study, Refractory RA with PD patients(n=19)	Subgingival plaque, serum and synovial fluid by PCR	*P. intermedia* (89.4% and 73.6%) and *Porphyromonas gingivalis* (57.8% and 42.1%) were the species most frequently detected in subgingival dental plaque and synovial fluid	(94)
Reichert et al, Germany, 2013	RA patients >18 years of age	Pregnant, and had taken antibiotics in the past 3 months or undergone subgingival scaling procedures 6 months prior to the examination	Cross-sectional study, RA patients (n=42), and non-RA subjects (n=114)	Subgingival plaque by 16S rRNA gene amplicon sequencing and synovial fluid by PCR	In RA patients, *P. gingivalis* DNA is more frequently detected in synovial fluid compared to controls	(92)
Zhao et al, China, 2018	Patients with diagnosed RA	Having chronic infectious disease history, experiencing an infection within 1 month before sampling, or receiving antibiotic therapy within 2 weeks before sampling	Cohort study, RA patients(n=125), OA patients(n=58)	Synovial tissue and synovial fluid, 16S rRNA gene amplicon sequencing	*Porphyromonas* and *Bacteroides* were present in all samples. *Veillonella dispar*, *Haemophilus parainfluenzae*, *Prevotella copri*, and *Treponema amylovorum* were more abundant in RA synovial fluid	(95)
Moen et al, Norway, 2006	Patients with diagnosed RA	None	Cross-sectional study, RA patients (n=16), psoriatic arthritis patients (n=14), and osteoarthritis patients(n=9)	Subgingival plaque, serum and synovial fluid by checkerboard DNA-DNA-hybridization	Higher variety and concentrations of oral bacterial DNAs were found in RA synovial fluid compared to serum. Exclusive species identified in RA synovial fluid included *S. intermedius*, *P. gingivalis*, *A. actinomycetemcomitans* and *F. nucleatum*	(91)
Totaro et al, Italy, 2013	Patients with diagnosed RA	None	Case-control study, RA patients(n =32), other arthritides patients(n =37), patients with active knee arthritis(n =69), healthy controlss(n =26)	Synovial fluid, synovial and tissue, peripheral blood and subgingival dental plaque by PCR	RA patients showed a higher prevalence of *P. gingivalis* DNA in synovial tissue compared to controls, especially among those with the HLA DRB1*04 allele.	(93)

These studies have established a chain of evidence for the translocation of oral bacteria to the joints, with particular emphasis on the colonization of *P. gingivalis* in synovial tissues, its intracellular localization potential, and its association with genetic susceptibility. These findings provide strong clues for its direct involvement in local joint pathology. However, the specific routes through which oral bacteria reach the joints, the extent of their contribution to arthritis pathogenesis, and the active components involved, such as intact bacteria, DNA, or metabolic products, remain to be fully elucidated.

## 4 Effects of periodontopathogen on experimental arthritis

Although many oral bacteria have been linked to RA, *P. gingivalis* is the most extensively studied species in terms of its impact on arthritis progression in animal models. As the predominant pathogen in subgingival polymicrobial communities associated with PD, *P. gingivalis* has been the focus of numerous experimental investigations in this field. Various arthritis models incorporating *P. gingivalis* infection have been employed to simulate its differential effects on RA. In these models, variations in immunization protocols, bacterial dosages, and routes of administration can markedly influence experimental outcomes. Moreover, the timing of arthritis induction relative to periodontal infection introduces additional complexity to experimental design, making reproducibility and interpretation particularly challenging. Twenty-three studies have examined the effect of *P. gingivalis* infection on the progression of RA using animal models (**Table 3**). Seventeen studies reported that *P. gingivalis* exacerbated arthritis, while four studies investigated the role of *P. gingivalis* combined with other oral pathogens, with three showing synergistic effects and one showing no significant effect on pristane-induced arthritis (98, 100, 101). Three reported synergistic effects, while one found no significant effect on pristane-induced arthritis (102). One study showed that oral inoculation with *P. gingivalis* led to the onset of seropositive arthritis, systemic inflammation, and bone erosion over an 8-month observation period (103). Additional study suggested that *P. gingivalis* failed to worsen arthritis but altered synovial proteome characteristics, suggesting that oral pathogens may contribute to the formation of particular disease subtypes (104). These studies had highly variable designs, using different *P. gingivalis* strains, models, and time points.

**Table 3:** Studies using animals on the association *P. gingivalis* – RA.

Author, country, year	Model	Species	Intervention	Duration	Results	Ref.
Kim et al, Republic of Korea, 2022	3 wk old male CIA mice	DBA/1J mice	*P. gingivalis*(ATCC33277), oral inoculation, Con: oral inoculation with PBS	3/wk, 3w, started at 14 days before the first immunization	*P. gingivalis* and *T. denticola* exacerbated arthritis+ increased serum RF with *P.gingivalis*	(63)
Sato et al, Japan, 2017	6 wk old male CIA mice	DBA/1J mice	*P. gingivalis*(W83),oral inoculation, Con: oral inoculation with PBS	2/wk, 5w, started at 1 days before the first immunization	*P.gingivalis* but not *P. intermedia* exacerbated arthritis+ increased serum IL-17 -gut microbiota changes, Th17 increased in MLN	(105)
Chukkapalli et al, USA, 2016	6 wk old male CIA mice	B10.RIII mice	Polybacterial mixture of *P. gingivalis* (FDC 381), *T. denticola*, and *T. forsythia*, oral inoculation, Con: oral inoculation with CMC	4 consecutive days/wk, 8w, started at 7 days before the first immunization	Polybacterial mixture exacerbated arthritis+increased serum MMP3; dissemination hematogenously to synovial joints with *P. gingivalis*	(98)
Yang et al, China, 2024	6 wk old male CIA mice	C3H/HeN mice	*P. gingivalis*(ATCC33277), oral inoculation, Con: oral inoculation with CMC	1 /w2d, 56d, started at 14 days before the first immunization	*P.gingivalis* exacerbated arthritis +increased serum ACPA- increased citrullinated protein in immune organs + altered immune cell subpopulations in spleen and lymph nodes	(106)
Zhou et al, China, 2021	5 wk old male CIA mice	DBA/1J mice	*P. gingivalis*(W83), oral inoculation, Con: oral inoculation with CMC/PBS	3/wk, 4w, started at 14 days before the first immunization	*P.gingivalis* increased arthritis and speed of RA onset, increased Th17 ^+^ Treg, down-regulated B10 cells	(107)
Hamamoto et al, Japan, 2020	Lamarin induced arthritis mice	SKG mice	*P. gingivalis*(ATCC33277 and W83), oral inoculation, Con: oral inoculation with CMC	2/wk, 6w, lamarin injection and *P. gingivalis* inoculation were started the same day	Joint destruction and gut microbiota dysbiosis/gut inflammation associated with *P.gingivalis* —gut bacteria transplants + increased citrullinated protein in joint and oral tissue	(108)
Yamakawa et al, Japan, 2016	6–8 wk old females lamarin induced arthritis mice	SKG mice	*P. gingivalis*(W83), intraperitoneal injections, Con: intraperitoneal injections with *E. coli*	1/wk, 6w, lamarin and *P. gingivalis* injection were started the same day	Joint bone destruction with *P. gingivalis* + increased MMP3 - enhanced osteoclastogenesis + more citrullinated protein in joint	(109)
Marchesan et al, USA, 2013	6 wk old male CIA mice	DBA/1J mice	*P. gingivalis*(W83), oral gavage, Con: oral gavage with CMC	1/d, 15d, started at 30 days before the first immunization	*P.gingivalis* decreased alveolar bone + increased arthritis incidence and severity- activated Th17-related pathways	(110)
Gully et al, Australia, 2014	6–8 wk old female CIA mice	BALB/c mice	*P. gingivalis*(W50) and *P. gingivalis* PAD-deficient, oral inoculation, Con: oral inoculation with CMC	2/wk, 7w, started at 44 days before the immunization	Reduced arthritis and periodontal inflammation with PAD-deficient strain of *P. gingivalis*	(111)
Maresz et al, Poland, 2013	6–8 wk old male CIA mice	DBA/1 mice	*P. gingivalis*(W50), isogenic PPAD-knockout strain and complement mutant, chamber inoculation, Con: chamber inoculation with sterilized of the same culture medium	2w, started at 14 days before the immunization	*P.gingivalis* increased arthritis and speed of RA onset -dependent on the expression of PPAD	(112)
Courbon et al, France, 2019	Female lewis rats	Lewis rats	*P. gingivalis*(W50), oral inoculation, Con: oral inoculation with gel	4/wk, 8 month, data not shown	*P. gingivalis* induced early arthritis and serum CCP	(103)
Jung et al, Republic of Korea, 2017	6 wk old male CIA mice	DBA/1J mice	*P. gingivalis*(W83 and 2561), oral inoculation and intraperitoneal inoculation, Con: oral inoculation and Intraperitoneal inoculation with CMC	2/wk, 9w, started at 7 days before the first immunization; started after the second immunization	*P. gingivalis* strain W83 not 2561 exacerbated arthritis before autoimmune reaction initiated	(113)
Sanda et al, USA, 2016	10–12 wk old CIA mice	*HLA-DR1* humanized C57BL/6 mice	*P. gingivalis*(W83), oral inoculation, Con: oral inoculation with CMC	1/d, 7d, started at 26 days after the immunization	Exposure of gingival tissues to *P. gingivalis* has systemic effects+increased ACPA	(114)
Muñoz-Atienza et al, UK, 2020	11 wk old male arthritogenic K/BxN serum induced arthritis mice	C57BL/6 mice	*P. gingivalis* (W50) and PPAD mutant strain, oral inoculation, Con: oral inoculation with CMC	1/w2d, 7d, started at 1 days before the immunization	Deletion of PPAD did not prevent *P. gingivalis*-mediated intestinal barrier breakdown and exacerbation of arthritis	(115)
Buschhart et al, Germany, 2020	8–12 wk old male CIA mice	DBA/1 and B10.Q mice	*P. gingivalis*(W83), oral inoculation, Con: oral inoculation with CMC	1/w2d, 14d, started at 31 days before the first immunization	*P. gingivalis* altered synovial proteome signature	(104)
Jeong et al, South Korea, 2018	5 wk old female CIA mice	DBA1/J mice	*P. gingivalis*(2561), oral inoculation, Con: oral inoculation with CMC	1/d, 15d, started at 15 days before the immunization	Anti-FimA Ab inhibited the exacerbation of CIA and PD	(116)
de Aquino et al, Brazil, 2017	18-22g weight male AIA(mBSA) mice	C57BL/6 mice and IL-17RA-KO	*P. gingivalis*(ATCC33277 ), oral inoculation, Con: oral inoculation with CMC	1/d, 5d, started at 1 days before the final immunization	*P. gingivalis* increased TNF, IL-17 and PMN accumulation in arthritic-dependent on IL-17RA	(117)
Munenaga et al, Japan, 2018	6–8 wk old female laminarin induced arthritis	SKG mice	*P. gingivalis*(W83), oral inoculation, Con: oral inoculation with CMC	1/w3d, 6w, started at 1 days after the immunization	*P. gingivalis* increased arthritis and bone destruction+elevated serum C5a	(118)
Peng et al, Taiwan, 2020	6–7 wk old male CIA arthritis	SD rats	Gingipain domains of *P. gingivalis* (ATCC33277), subcutaneous injections, Con: subcutaneous injections with PBS	1/d, started at 28 days before the immunization	Pre-immunization against gingipain domains alleviated arthritis severity and reduced MMPs	(119)

CIA: collagen-induced arthritis; MLN: mesenteric lymphocytes; CMC: carboxymethyl cellulose; B10: IL-10 producing regulatory B cells; Treg: Regulatory T Cell; AIA: antigen-induced arthritis.

Polymicrobial oral infections have been shown to accelerate experimental arthritis, leading to earlier disease onset and increased severity characterized by enhanced inflammatory cell infiltration and pannus formation. In addition to the well-established role of the red complex, a major pathogenic group in periodontitis (98), the combined infection with *P. gingivalis*, *Fusobacterium nucleatum*, and *A. actinomycetemcomitans* similarly promotes the onset and progression of RA (100). Interestingly, while this bacterial consortium exacerbates arthritis, it results in comparatively less alveolar bone loss in mice than individual pathogens, suggesting a complex interplay between local and systemic immune responses (100). Repeated oral inoculation with *P. gingivalis* and *Prevotella nigrescens* promotes RA through Th17-mediated immune responses, and their effect is directly correlated with the severity of joint bone erosion (101). However, not all oral bacteria aggravate experimental arthritis. For instance, periodontitis induced by *A. actinomycetemcomitans* does not affect the course of antigen-induced arthritis (AIA) in mice, whereas arthritis aggravates alveolar bone loss caused by this bacterium, primarily due to enhanced systemic inflammation and lymphocyte polarization (120). Notably, both anti-TNF-α and antimicrobial therapies can alleviate periodontitis in this context (120).

## 5 Systemic humoral immune responses to periodontopathogens in RA patients

### 5.1 Circulating antibodies against *P. gingivalis* and related bacterial components

Recent studies suggest that humoral immune responses targeting oral bacteria may play a role in the pathogenesis of RA (121). Infection with *P. gingivalis* elicits a host immune response characterized by the generation of specific antibodies, primarily targeting its bacterial components and major virulence factors, including arginine-specific gingipains (RgpA and RgpB) and PPAD (122). Multiple serological studies have consistently reported that individuals with RA exhibit significantly elevated levels of *P. gingivalis*-specific antibodies compared to healthy controls or first-degree relatives (123–126). The seroprevalence of these antibodies is also comparable between patients with RA (48.9%) and those with PD (52.7%) (83). Moreover, antibody titers have been shown to correlate with the severity of PD (127).

Antibodies targeting *P. gingivalis* are closely associated with key biomarkers of RA (**Table 4**). Among these, the association between serum *P. gingivalis* antibody titers and ACPAs has been one of the most robust and consistently observed findings across multiple studies (124, 128–132). Mikuls et al. reported that elevated *P. gingivalis* antibody titers were positively correlated with specific ACPA subtypes, particularly anti-CCP IgM and IgG2 (124). Similarly, another study observed that patients with subgingival *P. gingivalis* colonization and elevated antibody titers exhibited increased expression of selective ACPAs, including antibodies to citrullinated filaggrin (85). Additionally, *P. gingivalis* antibody titers have been shown to correlate with RF levels in patients with RA, particularly among those with moderate-to-high disease activity (126). Notably, individuals with RF-IgA levels exceeding 75 IU/mL exhibited a five-fold increase in *P. gingivalis* antibody titers (133). However, some studies did not observe a direct association between *P. gingivalis* antibody levels and RA disease activity (123, 126, 127), suggesting that these antibodies may play a more specific role in autoimmune mechanisms rather than contributing directly to systemic inflammation.

**Table 4:** Studies in humans on the association periodontal microorganisms *Porphyromonas gingivalis* and RA.

Author, country, year	Study: design, number of participants, groups	Samples	Analyzed variables	Results	Ref.
Kozhakhmetov et al, Kazakhstan, 2023	Case-sectional study, RA patients in female(n=75) and healthy volunteers ( n=114)	Scrapings were collected from the tongue, gums, tonsils, and plaque	Oral microbiota; laboratory variables: CRP, ACPA and RF RA clinical assessment: DAS28, TJC, SJC, VAS; assessments of the periodontal status	Low RA disease activity samples had more *Porphyromonas*; *Prevotella_9* positively correlated with serum ACPA and RF levels	(67)
de Smit et al, Holland, 2012	Case-control study, patients with RA(n=95), Non-RA controls(n=44), Healthy controls(n=36)	Serum and subgingival samples	IgA, IgG, and IgM antibodies to *P.gingivalis*; oral microbiota; laboratory variables: CRP, IgM-RF, ACPAs clinical RA assessment: DAS28, disease duration, smoking status, BMI, and RA medication; assessments of the periodontal status: DPSI score	RA patients with severe PD had higher IgG- and IgM-anti *P. gingivalis* titers than non-RA controls with severe PD, despite similar subgingival *P. gingivalis* levels	(84)
Svärd et al, Sweden, 2023	Case-control study, SARA study RA patients (n=196),Karlskrona study RA patients(n=132), and healthy controls (n=101)	Serum and saliva	serum IgG and IgA antibodies and saliva IgA antibodies to RgpB; laboratory variables: IgG and IgA ACPA in serum and IgA ACPA in saliva; clinical RA assessment:DAS28-ESR	The saliva IgA anti-RgpB antibody levels were significantly higher in RA patients compared to healthy controls and were associated with RA disease activity, but not with PD or serum IgG ACPA.	(137)
Kharlamova et al, Sweden, 2016	Case-control study, RA patients(n =1974),controls without RA(n =377), PD patients(n =65), and controls without PD(n =59)	Serum	Anti-RgpB IgG; laboratory variables: ACPA; PD assessments: CAL, BOP, probing depth; smoking, *HLA-DRB1* subtyping and genotyping	Anti-RgpB IgG had a stronger association with RA than smoking, with interactions between anti-RgpB, smoking, and *HLA-DRB1 SE* in ACPA^+^ RA	(138)
Mikuls et al, USA, 2009	Case-control study, RA patients (n=78), PD patients (n=39), and healthy controls (n=40)	Serum	Antibody to *P. gingivalis*, IgG anti-CCP antibody; laboratory variables: RF, CCP, CRP	Elevated *P. gingivalis* titers were common in RA and PD, correlating with increased CRP, anti-CCP-IgM, and IgG2 levels	(124)
Hitchon et al, Canada, 2010	Case-control study, RA patients (n=82) and their relatives (n=205) from North American Native (NAN), NAN individuals (n=47) and 60 non-NAN controls(n =60)	Serum	IgG anti-*P. gingivalis* lipopolysaccharides; laboratory variables: IgM- and IgA-RF, anti-CCP2; *HLA-DRB1* genotyping	Anti-*P. gingivalis* levels were higher in RA patients than in relatives and controls, and higher in ACPA-positive than in ACPA-negative RA patients	(125)
Rinaudo-Gaujous et al, France, 2019	Self-controlled study and case control study, RA patients(n=69), healthy controls (n=27), IBD patients(n=28), and SLE patients (n=35)	Serum	Antibodies against *P. gingivalis* and *P. intermedia*, anti-*P. gingivalis* and anti-*Escherichia coli* LPS-specific antibodies; laboratory variables: CCP2, RF, *HLA-B27*; clinical RA assessment: DAS28, TJC, SJC, disease duration, patient’s global assessment of disease activity, wrist X-rays clinical response to infliximab	Anti-CCP2 titers were associated with anti-*P. gingivalis* LPS-specific antibodies titers	(132)
Kononoff et al, Finland, 2017	RA(n=86), SpAs(n=91) and undifferentiated arthritis(n=80)	Serum	Antibodies against *P. gingivalis* and *A. actinomycetemcomitans*; laboratory variables: ACPA, RF, MMP-3; clinical RA assessment: DAS28, TJC, SJC, disease duration, hands and feet X-rays,	*P. gingivalis-*IgA was more frequent in ACPA-positive than ACPA-negative disease	(128)
Davison et al, UK, 2021	Self-controlled study, PD patients(n=42)	Serum and supragingival plaque	IgG antibody titers against *P. gingivalis*, *P. intermedia*, and *A. actinomycetemcomitans*; laboratory variables: anti-CCP antibody; PD assessments: plaque scores, bleeding scores, probing depth, CAL, periodontal epithelial surface area, and periodontal inflamed surface area	The presence, rather than the relative abundance of *P. gingivalis* in subgingival plaques had significantly higher anti-*P. gingivalis* IgG and ACPA titers	(139)
Bello-Gualtero et al, Colombia, 2015	Cross-sectional study, pre-RA patients(n=119), early RA patients (n=48), controls (n=119)	Serum and supragingival plaque	IgG1 and IgG2 against *P. gingivalis*, PD assessments: probing depth, CAL, BOP, plaque index, and plaque index; clinical RA assessment: DAS28-CRP and DAS 28-ESR, SDAI, HAQ, VAS; laboratory variables: RF, hsCRP, ESR, IgG/IgA ACPAs	In both pre-RA and early RA, *P. gingivalis*-specific IgG2 was associated with ACPAs	(131)
Mikuls et al, Omaha, 2014	Case-control study, RA patients(n = 287) and osteoarthritis patients controls (n = 330)	Subgingival plaques and serum	IgG antibody to *P. gingivalis*, *P. intermedia*, and *F. nucleatum*, *P. gingivalis*; PD assessments: CAL, BOP, probing depth; clinical RA assessment: DAS-28, radiological evaluation; laboratory variables: RF, anti-CCP2, hsCRP; *HLA-DRB1* status by single-nucleotide polymorphisms	The anti-*P. gingivalis* were association with anti-CCP-2 and RF, and select ACPAs were elevated in patients with subgingival *P. gingivalis* and higher anti-P*. gingivalis* antibody levels	(85)
Lee et al, South Korea, 2015	Cross-sectional study, RA patients(n=248) and healthy controls(n=85)	Sera	Anti-*P. gingivalis* and anti-ENO1 antibodies; clinical RA assessment: disease duration, morning stiffness, TJC, SJC,VAS, DAS28, X-rays of joints; laboratory variables: RF, ESR, CCP; clinical periodontal indices: GI, BOP, pocket depth, CAL	RA patients had higher anti-*P. gingivalis* antibody titers than controls, but these titers were not linked to RA disease activity	(123)
Laugisch et al,Switzerland, 2016	Cohort study, RA patients(n=52), non-RA patients(n=44)	Serum and gingival crevicular fluid	*A. actinomycetemcomitans*, *P. gingivalis*, *Tannerella forsythia*, *Treponema denticola*, and *P. intermedia* by nucleic-acid-based methods; citrulline and PPAD activity by the modified Boyde and Rahmatullah method; antibodies against RgpB, citrullinated and non-citrullinated forms of vimentin, fibrinogen, and enolase, and CCP antibodies by ELSIA;	PAD and PPAD activities within the periodontium are elevated in RA and non-RA patients with periodontitis	(87)
Quirke et al, UK, 2014	Case-control study, RA patients(n=80), PD patients(n=44) and controls (n=82)	Serum	The level of PPAD, C351A and RgpB; the immune response to autocitrullinated PPAD	Antibodies to PPAD were elevated in the RA sera compared with controls and specificity of the anti-peptidyl citrullinated PPAD response was confirmed	(140)
Sherina et al, Sweden, 2022	Case-control study, RA patients(n = 2,859) and controls(n = 372)	serum	Citrulline-Reactivity: IgG reactivity against citrullinated peptides derived from *P.gingivalis* PAD (CPP3) and human citrullinated peptides	Cross-reactivity between *P. gingivalis* CPP3 and human citrullinated peptides, and a CPP3+/CCP2+ clone, derived from an RA blood memory B cell, was identified	(141)
Konig et al, USA, 2014	Case-control study, RA patients(n=80), healthy controls(n=39)	Serum	The citrullination status and biology of PPAD in *P gingivalis* and anti-PPAD antibody response; IgG antibodies to citrullinated recombinant PPAD and unmodified recombinant PPAD; clinical RA assessment: DAS28; PD assessments: probing depth, CAL	PPAD from *P. gingivalis* is truncated and not citrullinated. Anti-PPAD antibodies do not correlate with anti-cyclic citrullinated peptide levels or disease activity in RA but are decreased in RA patients with PD	(142)
Shimada et al, Japan, 2016	Case-control study, RA patients(n=52), healthy controls(n=26)	Serum	anti-CCP IgG, anti-PPAD IgG and PAD-4; clinical periodontal indices: number of teeth present, probing depth, CAL, supragingival plaque accumulation; and BOP; clinical RA assessment: DAS-28; laboratory variables: RF, hsCRP, IL-6 and TNF-a	Anti-CCP IgG and anti-PPAD IgG levels were elevated in RA, with a positive correlation and a significant association between anti-PPAD IgG levels and RA	(143)
Manoil et al, Sweden, 2021	Cross‐sectional study, RA patients (n = 52), patients with Behcet's disease (n = 40), healthy control (n = 57)	Gingival crevicular fluid and Serum	quantification of 10 different bacteria; clinical RA assessment: DAS28; laboratory variables: ESR, CRP, RF-IgM, RF-IgA; PD assessments: pockets depth, CAL, BOP, PI	RA patients with RF-IgA levels above 75 IU/mL showed five times higher *P. gingivalis* levels, especially in those with DAS-28 scores over 3.2	(133)
Okada et al, Japan, 2011	Case-control study, RA patients(n=80), healthy controls(n=38)	Serum	IgG antibodies to *P. gingivalis*, *P. intermedia*, *A.actinomycetemcomitans*, and *Eikenella corrodens*; laboratory variables: anti-CCP antibodies and RF; clinical RA assessment: DAS28-CRP; PD assessments: number of teeth present, probing depth, CAL, BOP, supragingival plaque accumulation	Anti-*P. gingivalis* IgG responses are significantly associated with RA, showing a strong correlation with RF levels but not with anti-CCP antibody levels or DAS28-CRP in RA patients	(126)
Terato et al, Japan, 2018	RA patients with rapid radiographic progression(RRP)(n=54), RA patients with none RRP(n=101) and healthy volunteers(n=38)	Sera	Antibodies against bacterial pathogens: IgG and IgA antibody levels of *P. gingivalis*-LPS, *E. coli*-LPS and *Streptococcus pyogenes*-peptidoglycan polysaccharide; RA clinical assessment: DAS28-ESR, TJC, SJC, VAS, mTSS, the presence of osteitis and precise osteitis area; laboratory variables: CCP, ESR, CRP, MMP3, IgM-RF, lymphocytes, WBC, RBC, Hb, TNF-α, IL-6	IgA/IgG anti-*P. gingivalis*-LPS antibody ratio positively correlated not only with RF, ESR, CRP and DAS28-ESR in the RRP group	(135)
Castillo et al, Colombia, 2023	Cross-sectional observational study, RA patients(n = 143) and individuals without RA(n = 112)	Subgingival plaque and serum	Quantification *P. gingivalis*, anti-RgpA and anti-PPAD antibodies; clinical RA assessment: DAS28; laboratory variables: RF, hsCRP, ESR, antibodies to ACPA-IgG/IgA; periodontal assessments: plaque index, GI, BOP, pocket depth, CAL	The combination of anti-RgpA/anti-PPAD demonstrated high specificity (93.7%) and a positive predictive value (82.5%) for identifying individuals with RA	(134)
Seror et al, South France, 2015	Prospective cohort study, early RA patients (n=694), healthy controls (n=79), SS controls (n=54), PD controls (n=61)	Subgingival plaque and sera	IgG antibodies to LPS of *P. gingivalis* and the presence of *P. gingivalis*; laboratory variables: ESR, CRP, RF, ACPA and pro-inflammatory cytokines; clinical RA assessment: TJC, SJC, DAS28,HAQ, radiological evaluation; typing of *HLA-DRB1* shared epitope	Among non-smokers, higher anti-*P. gingivalis* antibody titers were associated with a higher prevalence of erosive changes	(136)
Takeuchi-Hatanaka et al, Japan, 2022	Case-control cross-sectional study, RA patients(n=82), polymyalgia rheumatica patients(n=38)	Serum	IgG titers against periodontal pathogenic bacterial antigens: IgG antibodies to *P.gingivali*s and *A. actinomycetemcomitans*; clinical RA assessment: RA therapeutic response, DAS28; laboratory variables: ESR, CRP, ACPA	RA patients with positive *P. gingivalis* antibodies initially had higher anti-CCP, RF, ESR, DAS28-ESR, and HAQ scores. After 12 months of treatment, they continued to exhibit higher disease activity	(129)
Arvikar et al, USA, 2016	Case-control study, early RA patients (n=50), late RA patients(n =43), other CTD patients (n =17), SLE patients(n =17), blood bank donors (n =53), age-similar healthy hospital personnel(n =19)	Serum	*P. gingivalis* antibody levels; RA clinical assessment: DAS28-ESR, HAQ, CDAI, physician's global disease score; laboratory variables: ESR, CRP, RF and anti-CCP antibodies	RA patients with positive *P. gingivalis* antibody responses exhibited significantly higher levels of anti-CCP, RF, and ESR, along with a tendency for higher DAS28-ESR and HAQ scores than those without the antibodies	(130)
Beyer et al, Norway, 2018	Cross-sectional study, RA patients(n=78)	Subgingival plaque	Oral microbiota; laboratory evaluation: ESR, CRP, ACPA and RF RA clinical assessment: disease duration of RA, MHAQ, DAS28, TJC, SJC and VAS; PD assessments: BOP, CAL, probing depth, accumulation of dental plaque, oral hygiene questionnaire	There was no association between RA disease parameters(DAS28, VAS,ESR, RF) and *P. gingivalis* except for CRP	(68)
Wan Jiun et al, Malaysia, 2023	Cross-sectional study, RA patients(n=100)	Serum and supragingival plaque	*P. gingivalis* bacterial load; laboratory variables: anti-CCP antibody; clinical RA assessment: DAS28; PD assessments: number of teeth, probing depth, CAL, BOP, and plaque index	The presence and the amount of *P. gingivalis* are not significantly associated with the anti-CCP antibody level	(86)

TJC, tender joint count; SJC, swollen joint count; VAS, visual snalog scale; HAQ, health assessment questionnaire; CDAI, clinical disease activity Index; MMP, matrix metalloproteinase; BOP, bleeding on probing;TNF-α, tumor necrosis factor alpha; mTSS, modified total Sharp score; SDAI, Simplified Disease Activity Index.

Serum antibodies against *P. gingivalis* demonstrate potential in the diagnosis of RA, as well as in predicting disease progression and treatment outcomes (**Table 4**). Double positivity for anti-RgpA and anti-PPAD antibodies in peripheral blood significantly enhances the diagnostic accuracy for RA, with a specificity of 93.7% and a positive predictive value of 82.5% (134). Two independent studies have implicated *P. gingivalis* in accelerating the severity and progression of joint damage in RA. In patients experiencing rapid radiographic progression (RRP), the IgA/IgG ratio of anti-*P. gingivalis* lipopolysaccharide (LPS) antibodies was closely correlated with disease activity parameters, including DAS28-ESR, RF, erythrocyte sedimentation rate (ESR), and CRP levels (135). Similarly, a large early RA cohort study found that non-smoking patients with elevated *P. gingivalis* antibody titers had a higher rate of bone erosion (47.5%) (136). Beyond diagnosis and progression, *P. gingivalis* antibodies may serve as prognostic indicators of suboptimal therapeutic response. Early RA patients who are seropositive for these antibodies tend to exhibit higher disease activity, impaired joint function, and elevated levels of anti-CCP, RF, and systemic inflammation. Even after 12 months of standard treatment, this subgroup continues to display a trend toward increased disease activity (130). Convergent evidence from a separate study involving 82 treatment-naïve RA patients revealed that those with higher *P. gingivalis* antibody titers showed a less favorable response to therapy after three months (129).

Although existing evidence suggests a role for *P. gingivalis* antibodies in RA, growing evidence indicates that these antibodies are more strongly associated with PD. This suggests that the observed link to RA may reflect the broader pathophysiological state of PD rather than the effect of a specific pathogen.

### 5.2 Circulating antibodies against other periodontal pathogens and related bacterial components

Beyond *P. gingivalis*, circulating antibodies targeting other periodontal pathogens and their virulence factors have also been implicated in the pathogenesis and clinical manifestations of RA, expanding our understanding of how humoral immune responses to periodontal infections may contribute to RA development.

Serological studies have identified a significant association between elevated anti-*A. actinomycetemcomitans* IgG titers and RA (126). Notably, all individuals infected with *A. actinomycetemcomitans* were found to be anti-CCP^+^ (144). The influence of *HLA-DRB1 SE* alleles on autoantibody positivity appears to be restricted to RA patients exposed to *A. actinomycetemcomitans (* 145). Leukotoxin A (LtxA), a key virulence factor secreted by *A. actinomycetemcomitans*, has recently been identified as a potential risk factor for the progression from arthralgia to RA (146). Anti-LtxA antibodies, including IgM, IgG, and IgA isotypes, can be detected across different stages of RA development, with significantly elevated IgM levels observed in both early and established RA patients (146). These antibodies are more prevalent among ACPA^+^ arthralgia patients and are associated with an increased likelihood of progression to RA (147). LtxA is thought to mimic membranolytic mechanisms involved in sustaining self-antigen citrullination in RA joints, inducing neutrophil morphological alterations and promoting neutrophil extracellular trap (NET) formation, which facilitates the release of highly citrullinated proteins in the oral cavity (145). Additionally, the N-terminal region of the DnaJ protein from *A. actinomycetemcomitans* elicits a specific immune response in RA patients, offering further insights into the disease’s etiology (148).

*P. intermedia* is a key periodontal pathogen implicated in chronic oral infection. Musculoskeletal ultrasound examinations have revealed that more than half of RA patients in clinical remission exhibit persistent synovitis. Notably, IgG titers against *P. intermedia* are significantly higher in patients with active RA and in those with ultrasound-detected synovitis (149). These findings suggest that *P. intermedia* may contribute to the chronic inflammatory state observed in RA. Through its enzymatic capacity to induce protein citrullination, *P. intermedia* may play a role in the generation of specific ACPA subtypes during the preclinical phase of RA. Prior to diagnosis, anti-*P. intermedia* antibodies are positively correlated with anti-CCP2, ACPA targeting vimentin, histones, and α-enolase, as well as with RF isotypes including IgA, IgG, and IgM (46). Further research has shown that *P. intermedia*-specific antibodies in gingival crevicular fluid are closely associated with ACPAs against citrullinated cytokeratin 13, a novel antigenic target with heightened antibody responses in RA patients that appears to lack cross-reactivity with antibodies directed against *P. gingivalis* gingipains (150). Interestingly, a large cross-sectional study (n = 33,994) found that elevated IgG responses to *P. intermedia* and Capnocytophaga ochracea were inversely associated with RF seropositivity (151). These findings imply that humoral immune responses to different periodontal pathogens may differentially influence RA-associated serological markers.

Circulating antibodies targeting other periodontal bacterial components, as well as the presence of these bacteria in the oral cavity, have also been linked to RA serological markers, although current evidence remains limited. *Haemophilus* species have been found to be depleted in both dental and salivary samples from RA patients and are negatively correlated with anti-CCP antibody levels (76). Subgingival colonization by *Aminipila butyrica* and *Peptococcus simiae* has been proposed to contribute to the generation of ACPAs (74). Additionally, *Lactobacillus salivarius* has been associated with elevated RA disease activity (74), further suggesting a potential role of specific oral microbiota in modulating systemic inflammatory states.

## 6 Molecular mimicry and immune cross-reactivity as core mechanisms of periodontopathogen-induced autoimmunity

### 6.1 Induction of citrullination and breaking immune tolerance

One of the central pathogenic mechanisms of RA is the breakdown of immune tolerance to citrullinated proteins, which involves increased production of citrullinated antigens and enhanced autoimmune responses against these antigens, particularly ACPA. Among various periodontal pathogens, *P. gingivalis* has drawn considerable attention due to its unique ability to induce citrullination. In animal models, oral administration of *P. gingivalis* has been shown to significantly elevate serum ACPA levels, accompanied by the detection of abundant *P. gingivalis* components and citrullinated proteins in the ankle joints (109). Upregulation of citrullinated protein expression has also been observed in multiple organs (108, 114). Moreover, a marked increase in the proportion of Tfh-gcB cell subsets responsible for autoantibody generation has been reported in lymphoid tissues (106). The contribution of *P. gingivalis* PPAD to citrullination has been demonstrated using gene manipulation approaches. Notably, wild-type *P. gingivalis*, but not PAD-deficient strains, induced the development of ACPA (108, 111) and production of citrullinated proteins (108, 112, 113), underscoring the mechanistic role of PPAD in linking *P. gingivalis* infection to RA pathogenesis. However, opposing evidence suggests that PPAD may not serve as the initial cross-reactive target for ACPA induction, as its absence does not prevent *P. gingivalis*-induced intestinal barrier impairment or exacerbation of arthritis in certain models (115).

A key mechanism by which PPAD induces host immune responses is enzymatic mimicry. This hypothesis, first proposed in 2009, suggests that *P. gingivalis* infection and PPAD-mediated citrullination of human antigens can trigger the ACPA response (39). Subsequent studies in RA patients have provided supporting evidence. It has been observed that peripheral plasmablasts in RA patients can produce ACPA, and approximately 63% of these antibodies cross-react with *P. gingivalis* outer membrane antigens and/or citrullinated enolase (152). Notably, in addition to C-terminal citrullination, *P. gingivalis* PAD exhibits prominent endocitrullination, targeting internal arginine residues (152). A 2020 study cloned and purified the PPAD enzyme (RACH2007-PPAD) from *P. gingivalis* strain CH2007 isolated from an RA patient and found that it could citrullinate internal arginine residues of major RA autoantigens such as fibrinogen and vimentin. Moreover, anti-RACH2007-PPAD antibody levels correlated with ACPA titers (153). More recently, a 2023 study proposed a possible mechanism by which persistent *P. gingivalis* exposure disrupts immune tolerance in RA. Repeated episodes of oral bacteremia in RA patients may result in widespread citrullination of oral bacteria, with subsequent translocation of citrullinated microbial antigens into circulation due to mucosal barrier breaches. This process activates proinflammatory monocyte subsets and inflamed synovial tissue, and further stimulates ACPA-producing B cells, thereby promoting affinity maturation and epitope spreading toward citrullinated human proteins (154).

Subsequent research has indicated that *P. gingivalis* primarily serves as an exogenous source of citrullinated antigens rather than modulating host-derived citrullination processes. Experimental evidence shows that when *P. gingivalis* is co-cultured with human peripheral blood mononuclear cells and macrophages, PPAD activity is detected exclusively in mononuclear cells exposed to the bacteria, leading to increased extracellular citrullination. In contrast, the expression of endogenous PAD enzymes and intracellular citrullination within monocytes and macrophages remains unaffected by *P. gingivalis* exposure (155).

### 6.2 Immune evasion

*P. gingivalis* employs various mechanisms to evade the host’s innate immune defense. *P. gingivalis* evades the immune response through PPAD-mediated citrullination by: 1) decreasing levels of neutrophil-derived proteins associated with phagocytosis and the antimicrobial activity of histone H2; 2) citrullinating histone H3, which facilitates *P. gingivalis* in evading NETs; 3) citrullinating the lysozyme-derived cationic antimicrobial peptide LP9, thereby limiting its antimicrobial activity (156). However, this immune evasion has been primarily observed in the oral cavity, and further confirmation in the context of RA is needed. Additionally, *P. gingivalis* facilitates immune evasion through A-type lipopolysaccharide (A-LPS), which assists in the localization of PPAD to Outer membrane vesicles (OMVs). OMVs are secreted by *P. gingivalis* as spherical bilayer structures made up of outer membrane proteins, phospholipids, DNA, lipopolysaccharides and segments of the periplasm (157). These vesicles package and transport bacterial virulence factors, including PPAD, to various host tissues. A-LPS modification not only localizes PPAD to OMVs, but also protects PPAD from proteolytic degradation, thus facilitating its “protective secretion” (158). Although PPAD is not essential for bacterial biofilm formation, or for the attachment and penetration of gingival epithelial cells by *P. gingivalis*, it functions as a powerful regulator of the immune response by enhancing the expression of genes related to cytokines (159).

### 6.3 Modulation of host immune responses

Exposure to *P. gingivalis* in gingival tissue exerts systemic effects by activating the host immune response. It may accelerate arthritis progression in CIA mice by mobilizing dendritic cells, macrophages, and neutrophils to the joints (116). Oral infection with *P. gingivalis* in CIA mice not only increases arthritis scores but also elevates serum RF levels (63). Chronic oral colonization with *P. gingivalis* prior to arthritis induction enhances immune activation that favors Th17 cell responses (107, 110, 114). Studies using IL-17RA knockout mice have confirmed that the exacerbating effect of *P. gingivalis* on arthritis is dependent on the Th17/IL-17 signaling pathway (117). Interestingly, *P. gingivalis* can also compromise the intestinal barrier and alter gut microbiota, thereby contributing to systemic inflammation and further aggravating arthritis (105, 108). These effects are partially mediated by increased serum IL-17 levels and a higher proportion of Th17 cells in mesenteric lymph nodes (105). In addition, downregulation of IL-10–producing regulatory B cells has been proposed as a key mechanism by which *P. gingivalis* promotes RA, since other immunosuppressive cells such as Tregs and myeloid-derived suppressor cells (MDSCs) are upregulated rather than suppressed (107). It is noteworthy that experimental arthritis does not affect the degree of *P. gingivalis*-induced alveolar bone loss (117).

### 6.4 Effects on promotes bone destruction and cartilage damage

Osteoclast overactivation and differentiation are the primary mechanisms underlying articular bone erosion in RA. *P. gingivalis* is a key contributor to bone loss associated with inflammation. Toll-like receptor (TLR) signaling modulates both inflammation and bone metabolism, whereas the receptor activators of nuclear factor kappa-B(NF-κB) ligand (RANKL) and its receptor, RANK, serves as a crucial regulator of osteoclast activation and bone remodeling processes. In isolated cultures of mouse parietal bone, *P. gingivalis*-LPS, via TLR2, promotes the formation of differentiated, mature osteoclasts, leading to bone loss. This process is primarily dependent on elevated RANKL levels (160). Subsequent studies revealed novel crosstalk between TLR and RANKL signaling cascades in the process of osteoclastogenesis. *P. gingivalis* modulates differently RANKL-induced nuclear factor of activated T-cells 1 (NFATc1) and cellular proto-oncogene Fos (c-Fos) expression, contingent upon the differentiation stage of osteoclast precursors. It inhibits RANKL-driven osteoclastogenesis at initial stages but promotes osteoclast maturation at later phases. TLR2/MyD88 is a central regulator of osteoclast differentiation by *P. gingivalis*, while RANKL reduces cytokine production triggered by *P. gingivalis* by down-regulating TLR/NF-κB and up-regulating NFATc1 (161). Mycorrhizal hairs, a major virulence factor of *P. gingivalis*, also contribute to osteoclast activation. The long-type FimA and short-type Mfa1 filaments are proteinaceous structures originating from *P. gingivalis (* 162). The study found that both Mfa1 and FimA hyphae promote the differentiation and activation of osteoclasts. Significantly, the effect of Mfa1 on osteoclast differentiation was intense than that of FimA, and Mfa1 promoted osteoclast differentiation and activation in osteoclast precursor cells stimulated by RANKL (163).

Infection of macrophages by bacteria triggers the release of pro-inflammatory cytokines, with TNF-α stimulating osteoclasts that differentiate bone marrow macrophages both directly *in vitro* and indirectly via osteoblasts. A study in an environment exposed to *P. gingivalis* highlights the critical role of TLR2 signaling in osteoclastogenesis. *P. gingivalis* promotes the increased expression of TLR2 on macrophage surfaces, which in turn increases TNF-α production and enables a functional response of these macrophages to reinfection. The macrophage reaction to *P. gingivalis* stimulation depends on TNF-α and is independent of RANKL, IL-6, and IL-1β (164). Conversely, an alternative investigation proposed that *P. gingivalis* directly promotes RANKL-triggered osteoclastogenesis in RAW264 subclonal mouse macrophages, with TNF-α not involved in this process (165).

Chondrocyte apoptosis can lead to cartilage damage, resulting in tissue degeneration and joint deformity. A study found that infection with *P. gingivalis* significantly increased both early and late apoptosis of human primary chondrocytes (166). This finding was corroborated by Pischon et al., who showed that *P. gingivalis* can localize within cells (167).

In conclusion, numerous *in vitro* experiments and animal studies have highlighted the potential of *P. gingivalis* to elicit changes associated with RA. In animal studies, *P. gingivalis* is often introduced into the oral cavity to observe changes in distal joints, thereby mimicking the clinical situation. Moreover, the experimental design, in which the control group is typically sham or bacteria-free, should be reconsidered to better assess the specific role of *P. gingivalis* compared to other periodontal pathogens.

## 7 Preventive aspects from the microbiological point of view

The essence of establishing proof-of-concept hinges on tackling the underlying causative factor with treatments like nonsurgical periodontal treatment (NSPT), which involves providing oral hygiene guidance and mechanically removing microbes from tooth surfaces both above and below the gingival margin. Currently, there is no consensus on the common management strategies for periodontitis and rheumatoid arthritis. Studies have found that NSPT relieves inflammation for 6 weeks to 6 months and reduces DAS28, VAS, SJC, TJC and improves clinical periodontal parameters (168, 169). The meta-analysis showed that, beyond enhancing the management of disease activity in RA, NSPT alleviated the systemic biomarker serum CRP among individuals with RA. It also exhibited a tendency of decreased ESR, morning stiffness, RF, TNF-α, and IL-6 (170). Therefore, promoting and developing NSPT for RA patients should be prioritized. However, due to issues such as selection bias and frequent changes in RA medication, there remains a pressing need for well-designed studies to further investigate this topic. At present, it remains challenging to definitively assess the effect of periodontal treatment on RA. Nonetheless, the promotion and development of NSPT for RA patients should not be overlooked.

Another approach targets the virulence factors of microorganisms. FimA, an important bacterial protein of *P. gingivalis*, demonstrated the ability to minimize alveolar bone loss and arthritic bone destruction when preincubated with FimA antibody. It also lowered the adhesion and aggregation of bacteria on gingival and synovial fibroblasts derived from humans (116). A distinct amino acid sequence within the catalytic domain of RgpA exhibits homology to the sequence of type II collagen. Pre-immunization rats with the purified recombinant structural region of RgpA similarly reduced joint inflammation and bone erosion (119). Targeting key virulence factors of *P. gingivalis* through preimmunization or small molecule inhibitors have the potential to diminish the likelihood of pathogens spreading to distal tissues and decrease their ability to trigger or exacerbate pathological changes in RA.

Recent studies have shown that DMARDs can improve clinical parameters of PD while treating RA. To eliminate the potential confounding influence of patients adopting proper oral hygiene habits, the investigators selected a 4-week follow-up period. Following 4 weeks of treatment with methotrexate (MTX), hydroxychloroquine (HCQ), and sulfasalazine (SSZ), patients diagnosed with RA and comorbid periodontitis exhibited improvements in CAL and probing depths when compared to periodontally healthy subjects. Moreover, MTX alone reduced probing depth after 3 months in contrast to the combination of MTX with SSZ and HCQ. Other drugs, including the JAK-inhibitor baricitinib, tocilizumab, rituximab, and anti-TNF-α monoclonal antibodies, also provide similar remission in PD (171). Paradoxically, some studies have questioned the treatment effectiveness of DMARDs in PD. Treatment with synthetic DMARDs, alone or in combination with another synthetic or biologic DMARD, for 6 months did not significantly improve periodontal parameters, nor did it affect the presence of *P. gingivalis (* 172). In contrast, no improvement in the periodontal inflammatory surface area or in anti-*P. gingivalis* antibody levels was observed after 2 months of treatment with MTX or 3–6 months of treatment with anti-TNF-α antibody (173). The heterogeneity of periodontal parameters, variations in monitoring period duration, and the small quantity of randomized controlled trials with diverse DMARDs make it difficult to identify the most effective DMARD for treating PD. This requires further investigation.

Plant-derived products have been proposed as potential modulators of both microbiota and host responses. Curcumin, the primary curcuminoid in turmeric, is an effective antimicrobial, antioxidant, and anti-inflammatory agent against *P. gingivalis* infections and biofilm formation. It suppresses the pro-inflammatory response of Th17 cells and enhances the immunoregulatory functions of Tregs, yielding a dual therapeutic effect (174). Clinically, topical curcumin has been shown to reduce microbial load in patients with chronic PD (175). Curcumin can inhibit *P. gingivalis* biofilm formation by more than 80%, even at very low concentrations (20 μg/mL) (176).

## 8 Conclusion and outlook

An increasing body of preclinical animal studies and epidemiological research supports a close association between RA and the oral microbiome. This review explores the role of oral pathogens in the initiation and progression of RA, and highlights the presence and potential effects of various pathogenic species in the oral microbiota of RA patients. Most clinical studies have found that dysbiosis of the oral microbiome is already evident during the preclinical phase of RA. Pathogenic bacteria and their virulence factors may enter systemic circulation, elicit immune responses, and be detectable through the identification of bacterial DNA in peripheral samples. The review also analyzes key virulence factors of oral microbes, their pathogenic mechanisms, and their impact on host immune responses. Notably, *P. gingivalis* infection appears to increase the risk of developing RA and may reduce the therapeutic efficacy of DMARDs. However, most clinical studies are cross-sectional in nature and are subject to heterogeneity and confounding factors, making it difficult to establish definitive causal relationships. Identifying reliable microbial biomarkers for RA diagnosis or prediction remains a major challenge. In contrast, animal studies have provided stronger evidence that *P. gingivalis* and its major virulence factors accelerate the progression of experimental arthritis. These pathogenic effects are primarily mediated through four mechanisms (1): protein citrullination, (2) immune evasion, (3) modulation of host immune responses, and (4) promotion of bone destruction. It is important to note that current studies predominantly focus on the role of a single bacterial species in RA pathogenesis. Given that periodontitis is a polymicrobial and chronic inflammatory condition, future research should also investigate how microbial consortia contribute to RA development.

The unique pathogenicity of *P. gingivalis* remains a subject of ongoing debate. Although experimental animal models have demonstrated RA-related alterations following *P. gingivalis* exposure, the complexity of human disease makes the available evidence inconsistent, and the observed associations do not establish causality. Since RA primarily affects the joints, human studies, due to ethical and humanitarian considerations, are largely limited to analyses of the oral cavity and peripheral blood. As a result, there are limited opportunities to directly investigate changes at the primary site of disease. Although several studies have detected periodontal pathogens in synovial fluid and tissue, the specific pathogenic features, migration pathways from the oral cavity to the joints, and key molecular mediators involved in this process remain to be fully elucidated.

In conclusion, the roles of PD, *P. gingivalis*, and other periodontal pathogens in the pathogenesis of RA remain incompletely understood. The underlying mechanisms by which non-surgical periodontal therapy improves RA outcomes also require further clarification. Well-designed clinical trials with large sample sizes and extended follow-up periods are needed to provide more robust evidence. In the future, rigorous, standardized, and large-scale prospective studies, along with in-depth mechanistic investigations, are essential to clarify the causal relationship between oral pathogens and RA. Moreover, exploring interventions targeting the oral microbiome may offer valuable insights for RA prevention and management, potentially contributing to personalized therapeutic strategies.
